# Assessment of disease-free survival in patients with laryngeal squamous cell carcinoma treated with radiotherapy associated or not with chemotherapy

**DOI:** 10.1590/S1808-86942010000200013

**Published:** 2015-10-19

**Authors:** Helma Maria Chedid, Carlos Neutzling Lehn, Abrão Rapoport, Ali Amar, Sérgio Altino Franzi

**Affiliations:** Master's degree in health sciences, graduate course of the Hospital Heliópolis (Heliopolis Hospital). Assistant in the otorhinolaryngology and head & neck surgery department of the Hospital Heliópolis; Doctorate in medicine, UNIFESP. Head of the otorhinolaryngology and head & neck surgery department of the Hospital Heliópolis; Adjunct professor at the Sao Paulo University, Coordinator of the graduation course on health science, Hospital Heliópolis; Doctorate in medicine, UNIFESP. Assistant in the otorhinolaryngology and head & neck surgery department of the Hospital Heliópolis; Doctorate in medicine, Sao Paulo University (USP). Assistant in the otorhinolaryngology and head & neck surgery department of the Hospital Heliópolis. Hospital Heliópolis (Heliopolis Hospital)

**Keywords:** carcinoma, laryngeal neoplasms, disease-free survival, transitional cells, radiotherapy

## Abstract

In early stage (I and II) laryngeal squamous cell carcinoma, both surgery and radiotherapy results in significant local and regional control. In advanced tumors (III and IV), radiotherapy alone has local-regional control rates of 32-43%.

**Aim:**

To assess disease-free survival in SCC laryngeal carcinoma patients submitted to radiotherapy alone and/or associated with chemotherapy.

**Materials and Methods:**

Retrospective study involving 84 cases of laryngeal SCC treated with radiotherapy or chemotherapy together with radiotherapy. Fifty-three cases were treated with intension to cure and 31 because of impossibility to resect the disease. As to clinical stage (CS), 12 were CS I, 15 II, 21 III and 5 IV. In the second group, 11 cases were EC III and 20 IV.

**Results:**

Mean age was 60 years, 84.5% were men. Fifty-eight (69.1%) cases had complete response and 26 (30.9%) had persistent or residual disease. Five-year disease-free survival was of 42.5%; 62.5% of the patients with organ preservation indication and 9.75 in the group of irressecable disease.

**Conclusion:**

disease-free survival of those patients submitted to radiotherapy because of laryngeal SCC was of 62.5%

## INTRODUCTION

Patients with initial stage (I and II) laryngeal squamous cell carcinoma benefit from surgery and radiotherapy, showing significant rates of local and regional control and preserved speech, swallowing and breathing.^1^^,^^2^ The results in such early cases (stages I and II) are similar when using surgery or radiotherapy for local and regional control; rates are close to 100% for T1 tumors, and range from 55% to 75% for T2 tumors. Radical surgery is an option if tumors persist after radiotherapy, as may happen more commonly with T2 tumors.

The assumption that voice quality - especially in T1 tumors - benefits more from radiotherapy than from surgery is not completely true. Often after radiotherapy permanent fibrosis of the lamina propria may develop, which compromises the ability of the vocal fold to vibrate because of its need to be whole for proper function. Acceptable recovery of voice, however, is possible in poorly infiltrated T1 tumors treated surgically (cordectomy); in this situation, scarring results in a similar structure to the vocal fold, which may even vibrate. Nevertheless, the literature shows that better results in voice quality are seen in patients submitted to organ-sparing protocols, compared to surgery.^3^

Local and regional control of advanced squamous cell carcinoma (III and IV) is achieved in 32% to 43% with radiotherapy.^4^^,^^5^ However, adjuvant surgery to radiotherapy may significantly support local and regional control, particularly in patients with extensive primary tumors that involve the thyroid cartilage, soft tissues in the neck and the base of the tongue, and when there are metastases to neck lymph nodes.

The purpose of this study was to assess disease-free survival in patients with larynx squamous cell carcinoma undergoing conventional radiotherapy only or associated with chemotherapy.

## SERIES AND METHOD

A retrospective analysis was made of 84 patients with larynx squamous cell carcinoma admitted from January 1999 to December 2004, first treated with conventional radiotherapy. Inclusion criteria were previously untreated patients, where the indications for conventional radiotherapy were preservation of laryngeal function, refusal of surgery, extension of the primary tumor requiring reconstruction of patients in poor clinical conditions, and non-resectability. The exclusion criterion was the presence of distance metastases upon admittance.

The anatomical site of the disease was the glottis in 33 cases and the supraglottis in 49 cases. The anatomical site could not be defined in two cases because of extensive disease upon admittance.

Clinical staging was done according to the AJC-UICC (2002); there were 13 clinical stage I cases, 13 clinical stage II cases, 24 clinical stage III cases, and 34 clinical stage IV cases. The size of the primary tumor (T) and the presence of neck lymph nodes (N) were used to define two groups according to the indication of conventional radiotherapy: the first group consisted of patients that refused surgery and the candidates for preservation of laryngeal function; the second group comprised patients undergoing conventional radiotherapy because of disease extension associated with a poor clinical status and non-resectability (Table 1).

**Table 1:** Distribution of cases according to the indication of conventional radiotherapy and the clinical stage (TN).

Radiotherapy/T		N0	N1	N2	N3	Total
	*T1	12	2	0	0	14
	T2	13	0	2	0	15
Organ preservation	T3	12	7	3	0	22
	T4	2	0	0	0	2
	Total	39	9	5	0	53
	T1	0	0	0	0	0
	T2	0	0	2	4	6
Extension of tumor	T3	2	3	4	3	12
	T4	4	1	5	3	13
	Total	6	4	11	10	31

n= 84 patients; * 10 T1a, one T1b, and three cases with the epicenter in the supraglottis.

Conventional radiotherapy was done with a linear accelerator at a mean dose of 66.7 Gy (19 Gy to 74 Gy). The concomitant chemotherapy protocol recommends starting therapy on the tenth radiotherapy session with six cycles of cisplatin (CDDP) at 25 to 30 mg/m2/weekly. We indicate one to three cycles of cisplatin (CDDP) at 100 mg/m2 if neoadjuvant chemotherapy is initiated; in this case, a third of the dose is given in the first three days (D1, 2, 3), 450 mg/m2 (D1–5) 5-fluorouracil (5FU) and 135 mg/m2 (D1) paclitaxel.

The definition of local and regional control was absence of evidence of disease six months after the end of treatment. Persistence was considered as evidence of disease within the first six months after the end of treatment. Response to therapy was considered as the absence of evident lesions in the local and regional examination within 30 days after the end of radiotherapy.

The Kaplan Meier method was applied to study survival rates, and the Wilcoxon method was applied to investigate differences in survival. Patients that refused surgery were considered as candidates for organ preservation in the survival curves. The institutional review board of our institution approved this study (number 659).

## RESULTS

The mean follow-up of patients was 28.9 months; 10 patients (11.9%) were lost to outpatient follow-up within the study period. There were 74 male patients (84.5%) and 13 female patients (15.5%); the mean age was 60 years (37 to 78 years).

Fifty-four patients (64.3%) underwent radiotherapy exclusively, 24 patients (28.6%) underwent chemotherapy plus radiotherapy, and six patients (7.1%) underwent neoadjuvant chemotherapy plus chemoradiotherapy.

Conventional radiotherapy below 40 Gy was interrupted in four patients. In three cases this was because their clinical status worsened; a fourth patient abandoned therapy. At the end of radiotherapy, full local and regional response was observed in 58 patients (69.1%); the disease persisted in 26 patients (30.9%). These cases were distributed according to the epicenter of the tumor (in the glottis or supraglottis) where radiotherapy was indicated (Table 2).

**Table 2:** Distribution of the response to conventional radiotherapy, the tumor site and the indication for radiotherapy.

Site*/Radiotherapy		Control	Persistence	Total
	Preservation	17	7	27
Glottis	Extension	2	7	9
	Total	19	14	33
	Preservation	25	4	29
Supraglottis	Extension	12	8	20
	Total	37	12	49

n= 82 patients;

* two cases of extensive tumors, both with their tumors controlled at the end of radiotherapy

Table 3 shows the type of disease persistence (local, regional or local and regional) at the end of therapy (Table 3).

**Table 3:** Distribution of the type of persistence at the end of radiotherapy, according to the tumor site and the indication for radiotherapy.

Site/Response to radiotherapy			Persistence	
		Local	Regional	Local & regional
Glottis	Preservation	7	0	0
	Extension	6	0	1
Supraglottis	Preservation	2	2	0
	Extension	3	2	3

n= 26 patients.

**Chart 1:**
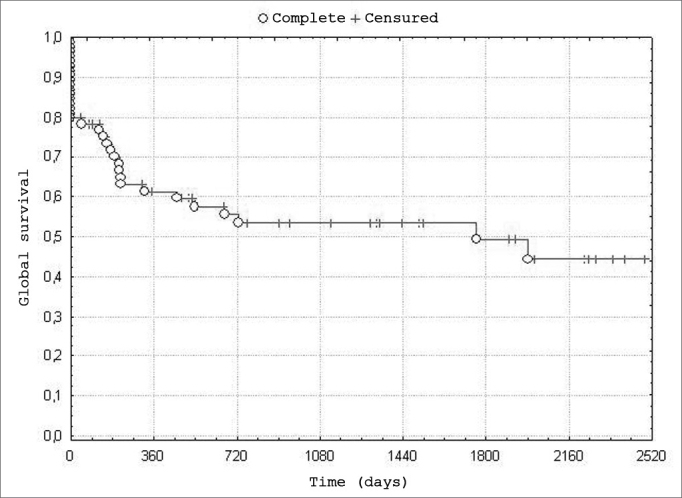
Distribution of global survival. - n=84 patients

**Chart 2:**
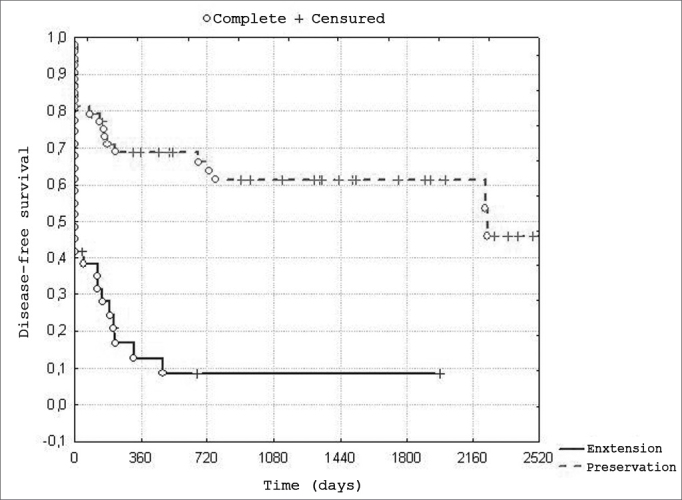
Distribution of disease-free survival according to the indication of - n= 84 patients, of which 53 underwent radiotherapy with preservation of the larynx, and 31 because of tumor extension or non-resectability.

The global survival rate among 84 patients was 47.5%; the 5-year disease free survival rate was 42.5%. The 5-year disease-free survival rate according to the indication of radiotherapy was 62.5% in organ preservation cases; it was 9.75% in patients irradiated due to disease extension or non-resectability (Chart 1, Chart 2).

**Chart 3:**
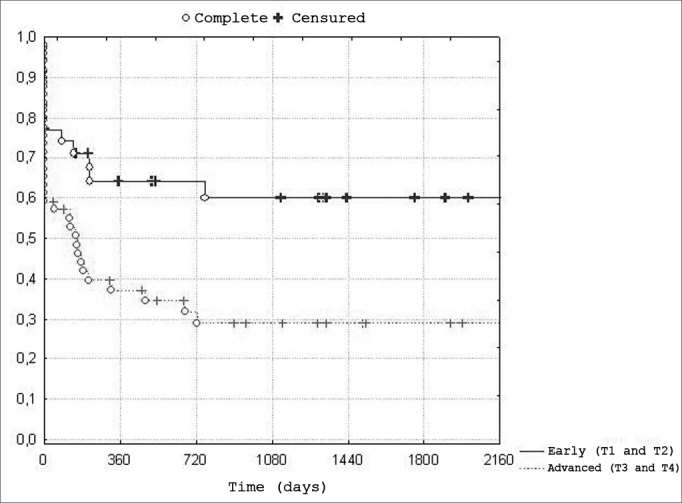
Distribution of disease-free survival according to the size of -n=84 patients, of which 35 with early tumors (T1 and T2), and 49 with advanced tumors (T3 and T4).

Early tumors (T1 and T2) and advanced cases (T3 and T4) were grouped, as seen below, to calculate disease-free survival in patients irradiated according to the size of the primary tumor (Chart 3).

The 5-year control rates of primary tumors (T) were 84% (T1), 44% (T2), 37% (T3) and zero (T4).

The 5-year disease-free survival was 38% in patients undergoing radiotherapy only, 49% when chemotherapy was added, and 45% in cases with induction chemotherapy for chemoradiotherapy (p=0.1). Seven clinical stage II tumor cases underwent radiotherapy only, and six such cases underwent chemoradiotherapy. Thirty-five patients in clinical stages III and IV underwent radiotherapy only; chemotherapy (concomitant) was added to the treatment of 24 such patients. Assessing concomitant chemotherapy in clinical stages II, III and IV and the type of indication of radiotherapy showed that therapy was indicated in 8/31 patients (25.8%) because of tumor extension or non-resectability, and in 21/40 patients (52.5%) due to organ preservation.

Twenty-six patients had persistent or residual disease at the end of radiotherapy; 18 of these were local, 4 were regional, and 4 were local and regional. Four of these patients underwent salvage surgery of the primary tumor - two subtotal laryngectomies with CHEP. In both case, lesions were previously T1 and T2. The other two patients underwent salvage surgery at another institution; one was a fronto-lateral laryngectomy (previously T1a) and the other was a total laryngectomy (previously T2).

There were 21 cases of local and regional recurrences; 10 were exclusively local, five were exclusively regional, five were local and regional, and one was local with a distance metastasis. Six patients underwent salvage surgery for treatment of the primary tumor - either total laryngectomy or pharyngolaryngectomy. Two of these cases were previously T1 lesions; the remaining cases were T3. Salvage neck dissection was carried out in two patients that had clinically metastatic lymph nodes on the first visit. Two patients refused salvage surgery.

Six patients had distance metastases; the lung was the site in all cases. Three cases presented a second primary tumor - two in the head and the neck.

By the last outpatient visit within the study period 29 patients had died because of the disease, 18 were alive but with local and regional disease and/or distance metastases, 5 had died due to other causes and 32 were alive with local, regional and distance control.

## DISCUSSION

Conventional radiotherapy aims to preserve laryngeal function in the treatment of early tumors (T1 and T2), similarly to partial surgery. However, the results are worse for local and regional control of advanced tumors (T3 and T4), especially if there are large lymph node metastases.^6^

Goepfert et al.^7^ showed that the results of radiotherapy are similar to those of surgery for supraglottic lesions that can be removed with partial laryngectomy; ranging from 88.5% for T1 lesions to 60% for T4 lesions. The M.D. Anderson Cancer Center, Houston (Texas), also demonstrated satisfactory results for local control of the disease in stages T1, T2 and selected T3 cases. In the beginning of the 1990s, local disease control rates were 100% (T1), 81% (T2), and 61% (T3) when using hyperfractionated radiotherapy.^8^

In the literature, local control of T1 glottic lesions reaches over 90%, varying according to involvement of one or both vocal folds.^9^^,^^10^ Thus, local control is decreased in lesions that involve the anterior commissure (92.3% 5-year disease-free survival rate) compared to lesions only in one vocal fold (85% 5-year disease-free survival rate).^10^

The 5-year local control rate among our T1 tumor cases was 84%. There were two cases in which the disease persisted after radiotherapy; these T1a lesions were treated with fronto-lateral laryngectomy and subtotal laryngectomy with CHEP. Local recurrence was diagnosed in three cases; all were glottic lesions, one of which was previously T1b. Two cases underwent total laryngectomy.

Local control of glottic T2 tumors with conventional radiotherapy ranged from 55% to 76%; this is lower than surgery as the first approach.^1^^,^^2^ A factor for poorer results in irradiated T2 tumors was the presence of vocal fold mobility; if there is mobility, local control rates are higher than 70%. On the other hand, radiotherapy is drastically less effective for lesions associated with decreased vocal fold mobility; a return to normal mobility after therapy is an independent prognostic factor regardless of the number of local recurrences.^11^ Other factors associated with a poorer prognosis is disease extension to the subglottis and the piriform recess.^2^ A published series has shown that the presence of an intermediate signal on the thyroid cartilage of patients with T2 tumors in magnetic resonance imaging highly suggested invasion of the thyroid cartilage and a worse prognosis.^12^

The main factor associated with a complete response to radiotherapy in supraglottic T2 tumors was tumor volume; best results are seen in superficial and lower volume tumors. Studies at Florida University have shown that local control was attained in 83% of tumors smaller than 6 cm^3^ as measured in computed tomography or magnetic resonance imaging. Local control was 46% in tumors larger than 6 cm^3^.^13^

In our study, 5-year local control of T2 tumors treated with conventional radiotherapy was lower compared to the literature. No association was seen with extension to neighboring sub-sites or decreased vocal fold mobility at the point when radiotherapy was indicated. However, the mean waiting time between the initial clinical visit and radiotherapy was 60 days (the distance between the radiotherapy center and the outpatient unit was often a difficulty for patients), which could result in disease progression and vocal fold paralysis, which would redefine the lesion as a T3 tumor at the beginning of therapy.

There were five cases of disease persistence at the end of conventional radiotherapy; two of these patients underwent salvage surgery (total laryngectomy and subtotal laryngectomy with CHEP. One in three cases of local recurrences underwent total salvage laryngectomy.

Local control of T3 tumors is poorer compared to initial surgery in advanced laryngeal tumors (III and IV), about 30% to 40%, and similar to our results.^4^^,^^14^ Harwood et al.^15^ showed that among radiotherapy-treated T3 lesions, worse results were seen in transglottic tumors. If transglottic tumors are excluded from our series, the local control rate reaches about 50%. Using hyperfractionated radiotherapy, the phase II ARCON study (accelerated radiotherapy, carbogen, nicotinamide) of 100 patients with advanced laryngeal tumors (T3 and T4) showed that 3-year local control was 80%.16 An issue is that T4 tumors extending to the muscles of the tongue base, soft tissues of the neck and major destruction of the thyroid cartilage were excluded. These factors probably account for decreased local control by radiotherapy in T4 tumors, because the indicated treatment is combined therapy (surgery and postoperative radiotherapy or radiotherapy and surgery); furthermore, most of these patients require tracheotomy and/or enteric tubes at the beginning of therapy.

In the present study, no persisting cases underwent salvage therapy; of seven local recurrences, four patients underwent total salvage laryngectomy. A disadvantage of radiotherapy in laryngeal T3 tumors is that it is harder to preserve function when indicating salvage surgery, especially in cases that previously would have undergone subtotal laryngectomy. In such cases, the best initial choice is subtotal laryngectomy. A further issue is the difficulty to diagnose local disease persistence on histology because of the side effects of radiotherapy - marked edema commonly seen in these patients, and necrosis of the thyroid cartilage, both of which delay salvage therapy. This factor may also be associated with disease progression and a poorer clinical status, making the disease non-resectable and/or inoperable.

On the other hand, supracricoid laryngectomy with CHEP as the initial therapy may lead to loss of function, such as aspiration and absence of decanulation. Chevalier et al.^17^ presented a 112-case series of laryngeal tumors initially treated with supracricoid laryngectomy with CHEP; 22 of these tumors were T3 stage. Two patients required a permanent tracheotomy postoperatively because of laryngeal stenosis, and underwent total laryngectomy.

Cases where subtotal laryngectomy with CHEP is indicated for T2 and T3 tumors, regardless of the neck lymph node status, surgery is the procedure of choice at our unit. Especially in T3 tumors, the prognosis of subtotal laryngectomy is comparable to total laryngectomy, with excellent functional results in most patients. Our institutional guidelines prioritize laryngectomy for the treatment of T3 tumors, even if the neck is N(0) and radiotherapy is offered as an alternative therapy.

In the early 1990s, the results of the Veterans Affairs Laryngeal Cancer Study Group showed that 38% were total laryngectomies in patients undergoing chemoradiotherapy; 56% were T4 tumors. The rate of salvage surgery was below 29% in tumors below stage T4.^18^ In that study, four patients with T4 tumors responded completely to conventional radiotherapy and did not require salvage surgery when there were local recurrences. However, most of the cases undergoing conventional radiotherapy were thus treated because the tumors were non-resectable or the disease was extensive and associated with poor clinical conditions. This scenario differs from some organ preservation protocols with resectable T4 disease, and those patients with lesions massively involving the thyroid cartilage and extending to soft tissues of the neck, which appear to respond better to surgery as the initial treatment; in such cases, preserving laryngeal function is uncommon in the patients at our unit.

Forastiere et al.^19^ showed that clinical stage II, II and IV patients undergoing chemoradiotherapy (78%) and induction chemotherapy (61%) had a higher rate of local and regional disease control compared to patients undergoing ionizing radiation alone (56%). Associating chemotherapy with radiotherapy undoubtedly yields superior local and regional control of the disease, which concurs with our findings in the present study. A higher number of indications for conventional radiotherapy alone are due to non-resectable tumors, extensive disease and organ preservation in early tumors (T1 and T2) with no palpable lymph nodes.

Among the indications of conventional radiotherapy in cases where regional disease persisted at the end of radiotherapy, no cases required neck dissection in our study. All patients had palpable neck lymph nodes (clinical stage N3) and/or non-resectable tumors that contraindicated initial salvage surgery.

## CONCLUSIONS

In this study of patients with larynx squamous cell carcinoma, in which conventional radiotherapy associated or not with chemotherapy was given, about 63.1% of cases with resectable disease and an indication for preserving organs had a 62.5% 5-year disease-free survival rate.
